# Defects in the Medial Entorhinal Cortex and Dentate Gyrus in the Mouse Model of Sanfilippo Syndrome Type B

**DOI:** 10.1371/journal.pone.0027461

**Published:** 2011-11-09

**Authors:** Kazuhiro Ohmi, Hui-Zhi Zhao, Elizabeth F. Neufeld

**Affiliations:** Department of Biological Chemistry, David Geffen School of Medicine, University of California Los Angeles, Los Angeles, California, United States of America; Nathan Kline Institute and New York University School of Medicine, United States of America

## Abstract

Sanfilippo syndrome type B (MPS IIIB) is characterized by profound mental retardation in childhood, dementia and death in late adolescence; it is caused by deficiency of α-N-acetylglucosaminidase and resulting lysosomal storage of heparan sulfate. A mouse model, generated by homologous recombination of the *Naglu* gene, was used to study pathological changes in the brain. We found earlier that neurons in the medial entorhinal cortex (MEC) and the dentate gyrus showed a number of secondary defects, including the presence of hyperphosphorylated tau (Ptau) detected with antibodies raised against Ptau in Alzheimer disease brain. By further use of immunohistochemistry, we now show staining in neurons of the same area for beta amyloid, extending the resemblance to Alzheimer disease. Ptau inclusions in the dentate gyrus of MPS IIIB mice were reduced in number when the mice were administered LiCl, a specific inhibitor of Gsk3β. Additional proteins found elevated in MEC include proteins involved in autophagy and the heparan sulfate proteoglycans, glypicans 1 and 5, the latter closely related to the primary defect. The level of secondary accumulations was associated with elevation of glypican, as seen by comparing brains of mice at different ages or with different mucopolysaccharide storage diseases. The MEC of an MPS IIIA mouse had the same intense immunostaining for glypican 1 and other markers as MPS IIIB, while MEC of MPS I and MPS II mice had weak staining, and MEC of an MPS VI mouse had no staining at all for the same proteins. A considerable amount of glypican was found in MEC of MPS IIIB mice outside of lysosomes. We propose that it is the extralysosomal glypican that would be harmful to neurons, because its heparan sulfate branches could potentiate the formation of Ptau and beta amyloid aggregates, which would be toxic as well as difficult to degrade.

## Introduction

The Sanfilippo syndrome comprises four mucopolysaccharide storage diseases (MPS III A-D) that have similar clinical phenotypes but are caused by different enzyme deficiencies in the lysosomal pathway of heparan sulfate degradation [1]. All four are characterized by profound mental retardation, behavioral problems, dementia, and death usually in the second decade, along with somatic manifestations that are milder than those seen in other MPS. Each of the MPS III subtypes is genetically heterogeneous, with some attenuated forms showing slower progression. We have concentrated on MPS IIIB, which is caused by mutation in the *NAGLU* gene and resulting deficiency of α-N-acetylglucosaminidase, and have made a *Naglu* knockout mouse by homologous recombination [2]. Biochemical and pathological findings plus a much shortened life span indicated that this mouse could serve as a model for the human disease in order to study pathogenesis and develop therapy.

Numerous studies of this mouse by our group and by others have addressed themselves to the neurologic problems of MPS IIIB. There is a strong inflammatory component in the brain disease, which is seen as activation of microglia [3], [4] with increased production of cytokines and chemokines [4], [5], up-regulation of immune-related genes [6], and even auto-immunity [7]. Astrocytes are also activated [8]. Alterations in vision and hearing as well as in circadian rhythm have been reported [9], [10], comparable to findings in the human disease. Both hypoactivity [2] and hyperactivity [11] have been noted in the open field test, but under different experimental conditions. The MPS IIIB mouse has been used for numerous therapeutic trials, including drugs [12], enzyme replacement [13] and gene therapy with various vectors [11], [14], [15], [16], [17], [18], [19].

We had observed that a number of pathological defects involving neurons were limited to a small areas of the brain of the MPS IIIB mice, mostly to layer 2 of the medial entorhinal cortex (MEC). The first defect to be observed in MEC was an increase in a lysosomal form of SCMAS (subunit c of mitochondrial ATP synthase) [20], suggesting autophagy or mitophagy and/or a general reduction in lysosomal proteolysis (SCMAS is a lipoprotein that is especially difficult to degrade and accumulates in a number of neurologic storage diseases [21]). Subsequently, we observed elevated cholesterol, GM3 ganglioside, ubiquitin and colloidal iron staining for glycosaminoglycans in the same cells [3], [20], as well as an increase in lysozyme and in hyperphosphorylated tau (Ptau) [22]. Ptau was also found in the dentate gyrus, which together with the medial entorhinal cortex is involved in learning and memory. The presence of Ptau is reminiscent of Alzheimer disease and other tauopathies, all of which lead to dementia [23].

The present study extends these findings to other proteins that are elevated in neurons of the MEC or dentate gyrus in the MPS IIIB mouse. These were detected by immuno-histochemistry at a sensitivity such that no staining was detected in a comparable area of unaffected control mice (*Naglu* +/−) or in an unaffected region of the MPS IIIB brain (represented by the lateral entorhinal cortex, LEC). The study seeks to understand the relationship of these secondary defects to the primary defect, failure to degrade heparan sulfate, as well as the relationship of the secondary defects to each other.

## Results

### MEC of MPS IIIB mouse is enriched in glypicans, heparan sulfate proteoglycans

**Figure 1:**
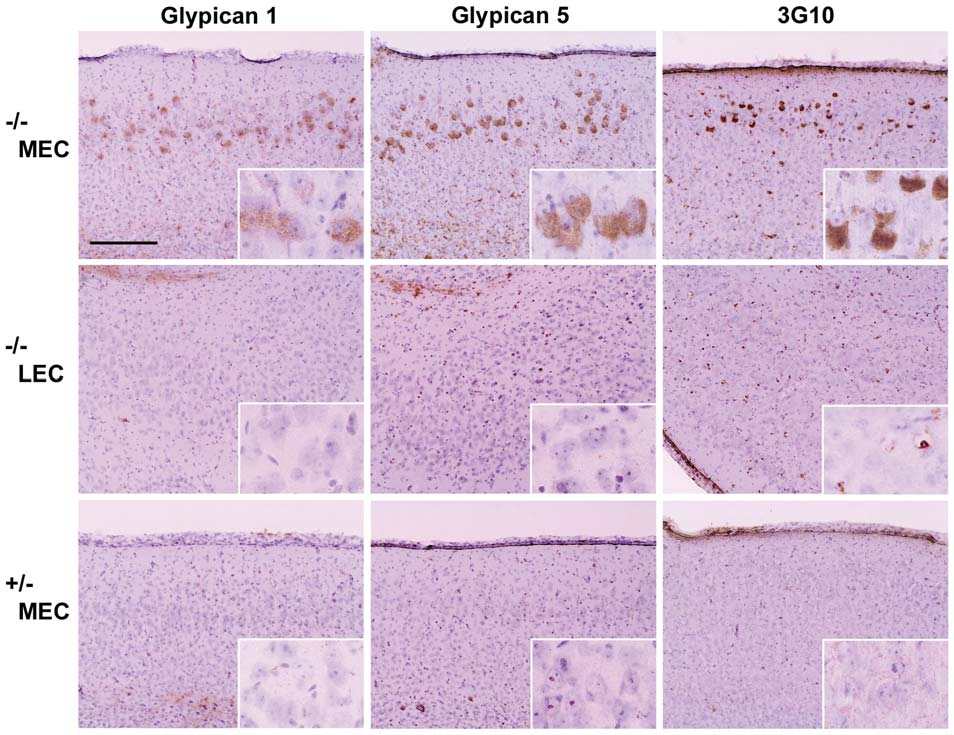
Elevated level of heparan sulfate proteoglycan in the MEC region of the MPS IIIB brain. Sections were stained, as indicated, with antibodies against epitopes in the protein core of glypican 1 and glypican 5 and against the carbohydrate neo-epitope 3G10 formed by pre-treatment of the proteoglycan with heparitinase. The top row shows prominent staining in the MEC region of 6 months old *Naglu* −/− mice, the middle row shows absence of such staining in the LEC region of these mice, and the bottom row, absence of staining in the MEC region of age-matched Naglu +/− (control) mice. The larger image was taken at 10X magnification, and the insert at 40X. The scale bar, 200 µm, applies to all figures in the panel.

Since disruption of the lysosomal pathway of heparan sulfate degradation is the primary cause of MPS IIIB, accumulation of heparan sulfate should be detectable by immuno-histochemistry. We were not successful in detecting heparan sulfate glycan with antibody HepSS-1. However, MEC neurons in the MPS IIIB brain showed strong staining with antibodies against glypican 1 and glypican 5, two heparan sulfate proteoglycans known to play an important role in the developing brain [24] (Fig. 1). Glypican staining was not observed in neurons from the LEC of the mutant mice nor from the MEC of control mice. There was no staining for glypican 2 (cerebroglycan), which is also important in early brain development (not shown); perhaps this proteoglycan is not expressed in the MEC at the ages tested (1, 3 and 6 months).

**Figure 2:**
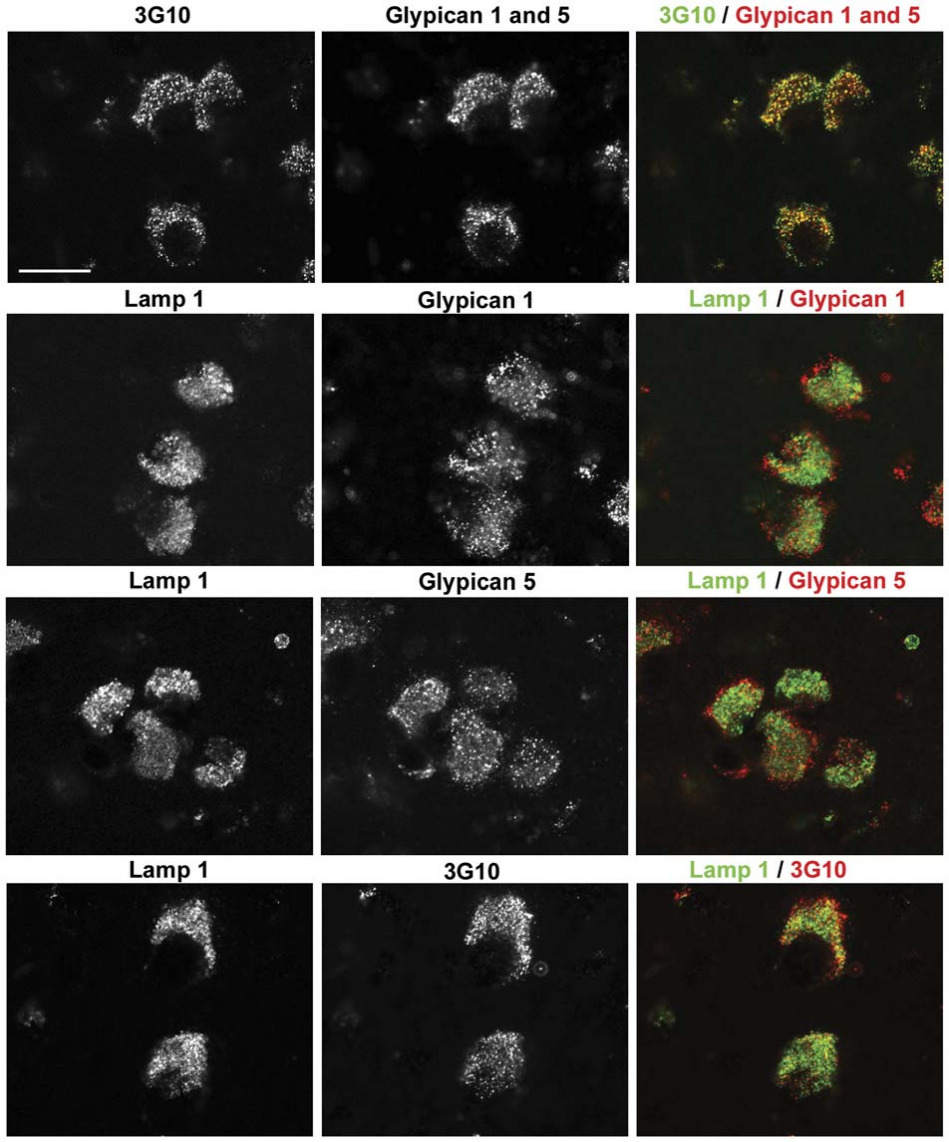
Co-localization of protein and carbohydrate moieties of heparan sulfate proteoglycans and only limited co-localization with lysosomes. The top row shows considerable but not complete co-localization of staining for the protein core of heparan sulfate proteoglycan (using a mixture of antibodies against glypican 1 and glypican 5) with staining for the carbohydrate neo-epitope 3G10. Subsequent rows show only limited co-localization of staining of antibody against Lamp1, a marker for lysosomes, and antibodies against glypican 1, glypican 5 and 3G10. But a considerable amount of the heparan sulfate proteoglycan, observed by antibodies against glypican 1 or 5 or the carbohydrate neo-epitope 3G10, is observed outside the area occupied by lysosomes. The mice in this experiment were 3 months old, except for the glypican 5 experiment which used 6 months old mice. The scale bar is 20 µm.

The epitopes of the glypican antibodies are in the protein moiety of the proteoglycan. The carbohydrate moiety was detected by pre-treating the sections with bacterial heparitinase (heparan sulfate lyase) and staining the resulting protein-linked carbohydrate stubs with the monoclonal antibody 3G10 [25]. Staining with 3G10 was most intense in neurons of MEC (Fig. 1) and to a lesser degree in neurons of the somatosensory cortex (not shown), but not of LEC. No staining was observed in neurons of control mice (*Naglu* +/−). The protein and carbohydrate stains were generally co-localized in neurons of MEC (Fig. 2, top row), but the presence of some 3G10 (green) staining not associated with glypicans 1 and 5 (red) suggests the existence of additional heparan sulfate proteoglycan(s), although it may also indicate carbohydrate stubs attached to glypican fragments that had lost their epitope due to partial proteolysis. No special biological significance is attributed to the presence of some glypican staining (red) without 3G10 staining (green), as it may be due to incomplete degradation of the carbohydrate chains by the heparitinase, so that the neo-epitope is not exposed.

Little glypican staining was seen in lysosomes, as shown by double staining with antibodies to Lamp 1 This was true whether the glypican was detected by the protein moiety (Fig. 2, rows 2 and 3) or the carbohydrate moiety (Fig. 2, bottom row). On the other hand, there was glypican clearly outside of lysosomes, as shown by the red stain.

### MEC of MPS IIIB mouse is enriched in Gsk3β

**Figure 3:**
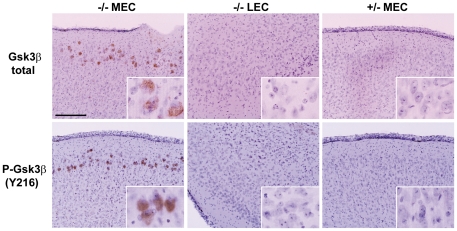
Elevated level of Gsk3β in the MEC region of MPS IIIB mouse brain. The left column row shows staining for total Gsk3β and for the phosphorylated form (P-Gsk3β Y216) in MEC of 6 months old MPS IIIB mice. The middle and right columns show absence of staining for these two markers in LEC of the MPS IIIB mouse and in MEC of the control (heterozygous) mouse, respectively. The scale bar is 200 µm.

The previous finding of Ptau in the medial entorhinal cortex and dentate gyrus of MPS IIIB mice [22] led to testing for Gsk3β, a candidate kinase. Both Gsk3β and P-Gsk3β (Y216), the form activated by phosphorylation on tyrosine 216 [26], showed intense staining in the MEC of the mutant mice, but not in the control area or in the control mice (Fig. 3).

**Figure 4:**
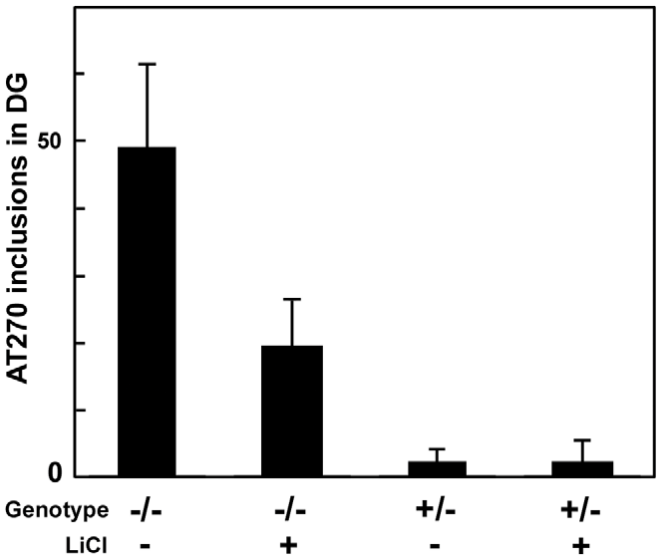
Effect of lithium administration on Ptau AT270 inclusions in the dentate gyrus. MPS IIIB (−/−) or control (+/−) mice were provided with LiCl in their chow, 3 g/kg, or fed regular chow, as indicated. Each group consisted of 8 male mice. Treatment was begun while the mice were nursing and ended with euthanasia at 3 months. The number of dentate gyrus inclusions staining with antibody AT270 was determined by counting 12 fields that include the dentate gyrus of the hippocampus. The bars represent the mean and standard deviation of the number of AT270 positive inclusions.

To determine whether Gsk3β was responsible for the formation of Ptau, lithium chloride, a selective inhibitor of Gsk3β [27] was administered to the mice in chow after weaning and the level of Ptau AT270 inclusions in the dentate gyrus was determined at the age of 90 days. To insure that the pups would be accustomed to the taste of lithium chloride, the nursing mothers were fed lithium chow from day P12. Fig. 4 shows that administration of lithium chloride in chow at a dose of 3g/kg reduced the number of AT270 positive Ptau inclusions in the dentate gyrus by 60% (p<0.01). A lesser amount (2 g/kg) had no effect (not shown), whereas a higher amount (4 g/kg) caused death of several pups and was discontinued. In contrast to the marked improvement in the dentate gyrus, we did not observe a change in MEC of immunostaining of Ptau AT100, beta amyloid, lysozyme, SCMAS, P-Gsk3β(Y216); however, immunostaining is not a quantitative test and might not have revealed a partial reduction.

### MEC of MPS IIIB mouse is enriched in beta amyloid

**Figure 5:**
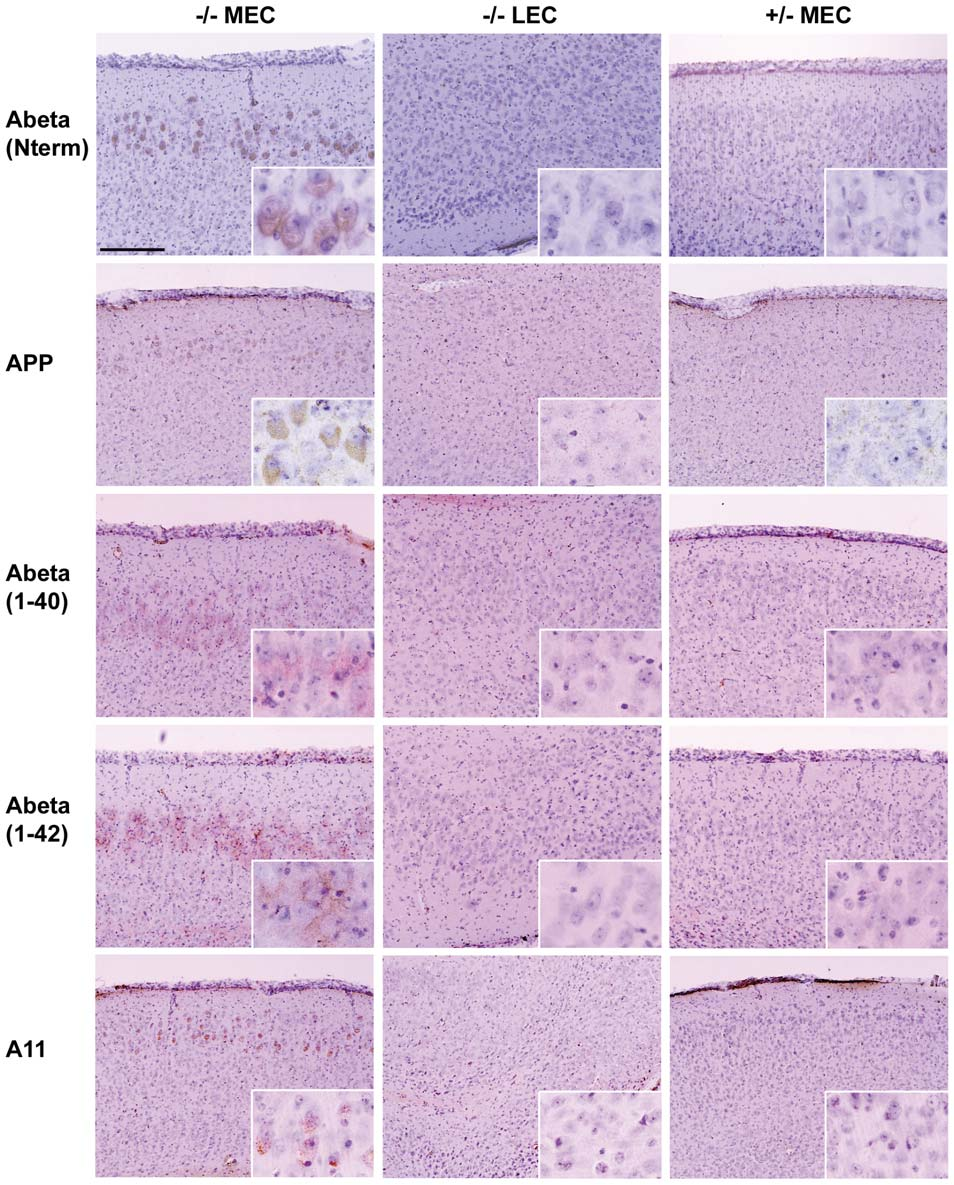
Elevated level of beta amyloid in the MEC region of MPS IIIB mouse brain. The left column shows staining in the MEC region of MPS IIIB (−/−) mice, with the middle and right columns showing a control region (LEC) in the same mice and the MEC region in control (+/−) mice, respectively. The mice were 3 months old for Abeta (N term), 6 months old for APP, Abeta 1–40, Abeta 1–42, and 10 months old for A11. The antibodies used, from top to bottom, were: polyclonal antibody to amino acids 1–14 of amyloid beta, polyclonal antibody to amyloid precursor protein (APP), monoclonal antibody to peptide Abeta 1–40, monoclonal antibody to Abeta 1–42, and polyclonal antibody to A11.

We found staining for beta amyloid in neurons of MEC of MPS IIIB mice by using antibodies raised against the 14 N-terminal amino acids, against the N-terminus of the amyloid precursor protein (APP), and against Abeta peptides 1–40 and 1–42; such staining was not found in the control region or unaffected mouse brain (Fig. 5).

Because beta amyloid is known to form oligomers, we tested reactivity with A11, an antibody that recognizes the oligomeric conformation of proteins independent of their sequence [28]. A11 staining was found in MEC but not in the control region or control mice (Fig. 5). However, the A11 immunoreactivity could be shared with oligomers of lysozyme, which is also elevated in MEC neurons [22].

### MEC of MPS IIIB mice is enriched in additional proteins

**Figure 6:**
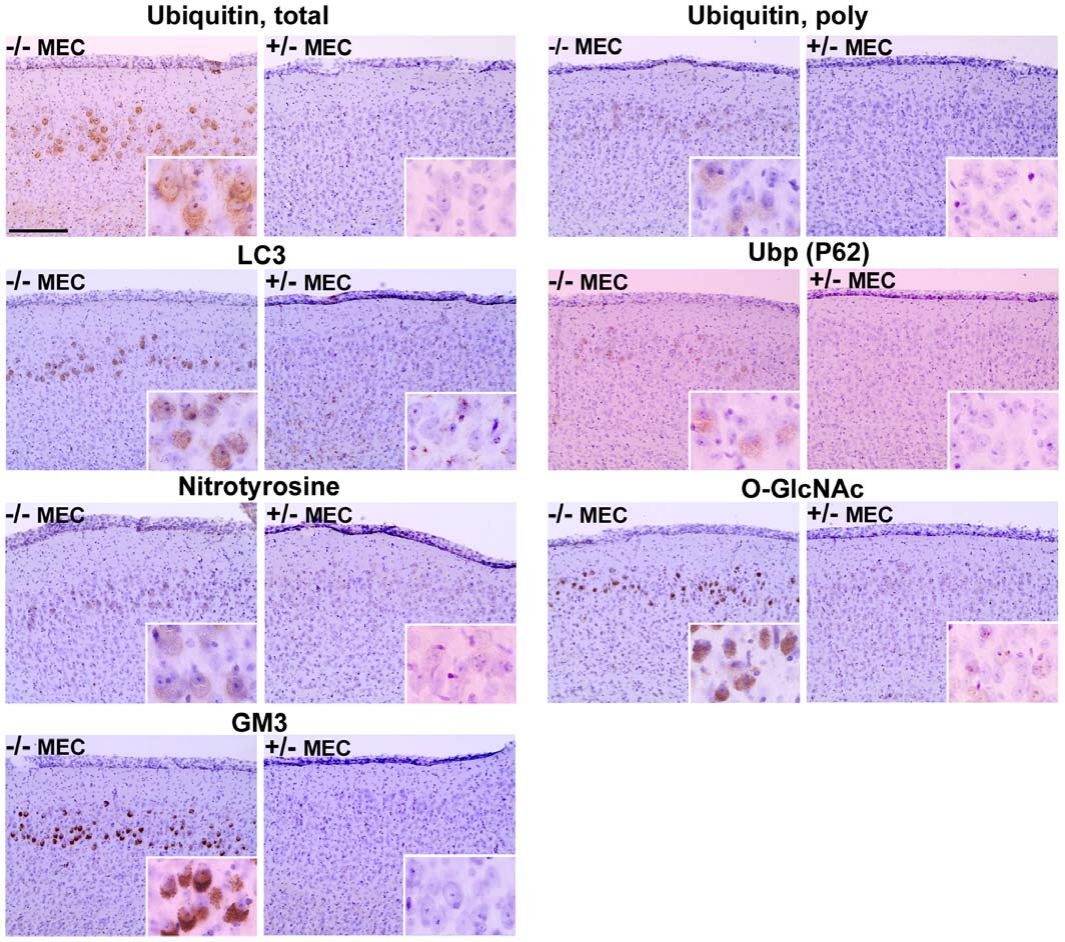
Elevated levels of additional proteins and GM3 ganglioside in the MEC of MPS IIIB brain. Staining performed with antibodies to the indicated substances was observed in the MEC region of 3 month-old MPS IIIB mice (for total ubiquitin and polyubiquitin) and 6 months for all others. Staining was not seen in the MEC region of age-matched control mice (*Naglu* +/−) nor in the LEC region of MPS IIIB mice (the latter not shown).

We have observed immunostaining of the MEC region of MPS IIIB mice for several other proteins (Fig. 6). These include proteins involved in autophagy: LC3, P62, and polyubiquitinated protein(s) [29]; nitrotyrosine-modified protein(s), a marker for oxidative stress, which has been reported in MPS IIIB brain [5]; and O-GlcNAc-modified protein(s), a marker for metabolic stress [30]. GM3 ganglioside and total ubiquitin are also shown in Fig. 6, because the earlier demonstration of their presence in MEC was indirect (i.e. localization in same cells as SCMAS [20]). The usual controls (−/− LEC and +/− MEC) showed no staining (not shown).

### MEC and dentate gyrus of MPS IIIA mice, but not of other mouse models of MPS, have the same defects as in MPS IIIB mice

**Figure 7:**
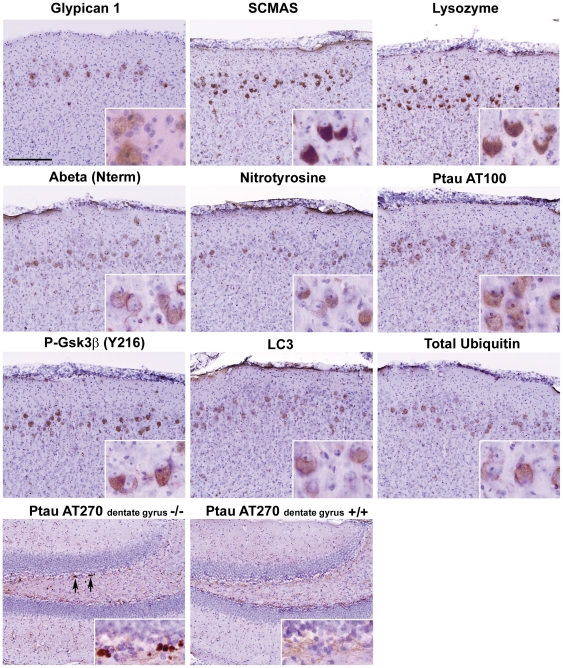
Elevated levels of various proteins in MEC and in dentate gyrus of MPS IIIA brain. Staining was performed with antibodies against the proteins shown in a 7 months-old MPS IIIA mouse brain. The first 3 rows are for the MEC region and the bottom row for dentate gyrus. Age-matched C57BL6 mice, used as controls, showed no staining in the MEC region (not shown). The dentate gyrus showed AT270 inclusions in MPS IIIA (−/−) mouse brain (arrows) but not in the C57BL/6 (control) brain (bottom row).

Immunohistochemical staining showed an elevated level of glypican 1 and of a number of proteins in the MEC of mice with MPS IIIA, which have a deficiency of sulfamidase, another enzyme of heparan sulfate degradation (Fig. 7). These other proteins were SCMAS, lysozyme, beta amyloid (amino acids 1–14), nitrotyrosine-modified protein, Ptau AT100, P-Gsk3β (Y216), LC3, and total ubiquitin. No immunostaining of these markers was observed in the control region or in MEC of age-matched C57BL6 mice (not shown). In addition, Ptau AT270 inclusions were seen in the dentate gyrus, within or at the border of the granular cell layer of the MPS IIIA mice but not of the C57BL6 mice (Fig. 7, bottom row). Thus the results in the MEC and dentate gyrus areas of the MPS IIIA brain were similar to those in the MPS IIIB brain for all markers tested.

The defects found in the MEC of mouse models of other MPS are shown in Table 1.

**Table 1:** Comparison of staining for markers in MEC of mice of different ages and with different mucopolysaccharide storage diseases.

MPS	IIIB	IIIB	IIIB	IIIB	IIIA	IIIA	I	I	II	VI
Age (mo)	0.5	1	3	6	4	7	6	12	7	12
Glypican 1	-	+	++	++	++	++	+	+	+	-
SCMAS	-	+	++	++	++	++	+	++	+	-
LC3	-	+	++	++	++	++	-	+/−	+/−	-
Nitroty	-	+	++	++	++	++	-	+/−	+/−	-
O-GlcNAc	-	+	++	++	+	nd		+	+	-

Staining: ++ intense to moderate.

+ weak.

+/- a few positive cells.

− not detected.

nd not determined.

There was only weak staining for glypican 1 in the MEC of mice with MPS I (deficiency of α-l-iduronidase) and MPS II (deficiency of iduronate sulfatase); staining for the secondary defects was likewise weak or marginal. There was no staining for glypican 1 in MEC of an MPS VI mouse (deficiency of N-acetylgalactosamine 4-sulfatase) nor any of the secondary defects.

### Defects in MEC are age dependent

No accumulation of glypican1 or of secondary markers was seen in the MPS IIIB MEC at the age of two weeks (Table 1). Weak staining for all the markers, indicating some accumulation, was seen at the age of 1 month, and became strong at the age of 3 months; little difference was observed between 3 and 6 months (Table 1).

## Discussion

This study and our earlier one [22] show three points of similarity between the pathology of the mouse MPS IIIB brain and that of the human brain with Alzheimer disease. First, the area most affected in mouse MPS IIIB, the medial entorhinal cortex, is the murine equivalent of the area in which Alzheimer disease starts, layer 2 of the entorhinal cortex [31]. Second, Ptau was detected in the MPS IIIB mouse brain with monoclonal antibodies raised against Ptau from Alzheimer disease brain. And last, beta amyloid, another hallmark of Alzheimer disease, has now been detected in the MPS IIIB mouse brain with four antibodies raised against different parts of beta amyloid or its precursor protein. However, there are major differences between the two diseases. The defects in the MPS IIIB brain do not extend much beyond the MEC, immunoreactivity against Alzheimer Ptau is limited to a few antibodies and all beta amyloid accumulation is intracellular. Unlike the case in Alzheimer disease, there are no extracellular plaques and no staining with thioflavin S in MPS IIIB. But as the toxic form of beta amyloid is now thought to be intracellular oligomers rather than the large aggregates in extracellular plaques [32], the absence of plaques does not diminish the possible role of beta amyloid in the pathogenesis of MPS IIIB. Neurons in the MPS IIIB MEC react with A11, an antibody that recognizes oligomers [28]; A11 may react with oligomers of beta amyloid and/or lysozyme, which is also present at increased levels in MPS IIIB [22].

The hyperphosphorylation of tau probably represents a late event in the pathogenic cascade leading to neurodegeneration. The AT270 inclusions of Ptau in the dentate gyrus require the action of Gsk3β, as demonstrated by the marked reduction of these bodies after treatment of the mice with lithium chloride (a specific inhibitor of Gsk3β from day 12 to day 90. It is probably the Gsk3β activated by phosphorylation on Y216 [26] that is responsible for the phosphorylation of tau; this form is elevated in the MPS IIIB brain, as is total Gsk3β. We did not attempt to determine the state of phosphorylation of the regulatory site, S9, because phosphorylation at that site is affected by anesthesia and post-mortem interval [33]. Since elevated P-Gsk3β was seen in MEC but not in the dentate gyrus, it is likely that the reaction product, Ptau, was transported from MEC to the dentate gyrus.

Other substances enriched in MEC suggest some metabolic patterns. Gangliosides, cholesterol and GPI-linked proteins cluster in lipid rafts [34]; therefore, the coexistent increase of GM3 ganglioside, cholesterol and glypicans in MPS IIIB MEC suggests enrichment of these substances in rafts on the plasma membranes of MEC neurons. The possible involvement of rafts in brain of MPS IIIA and IIIB mice was previously suggested [35]. LC3, P62 and polyubiquitin interact during the process of autophagy [29]; the elevated level of these proteins may therefore indicate autophagy that has not been completed. The failure of autophagosomes to fuse with lysosomes has been suggested to be a primary cause of pathology in lysosomal storage diseases [36]. The increase in total ubiquitin suggests inadequate proteasomal function, resulting in a backlog of ubiquitinated proteins, while the presence of SCMAS in lysosomes [20] suggests inadequate lysosomal proteolysis. The presence of nitrotyrosine-modified proteins suggests oxidative stress, which has been reported for the brain of MPS IIIB mice [5], while the increase in O-GlcNAc-modified proteins suggests metabolic stress [30]. The increase in O-GlcNAc-modified proteins indicates that the lack of heparan sulfate degradation and consequent lack of recycling of its constituent sugars does not cause a global reduction of N-acetylglucosamine residues on proteins, probably because sufficient N-acetylglucosamine can be made de novo by the glutamine: fructose-6-phosphate aminotransferase pathway.

What is the relationship of the primary defect (deficiency of an enzyme of heparan sulfate degradation) to these secondary defects? The secondary defects in MEC appear correlated with the increase in glypican 1, as seen by examining the brain of other MPS mouse models (Fig. 7 and Table 1). The accumulation of glypican 1 and the secondary defects in MEC of MPS IIIA mice are as prominent as those in the MPS IIIB mice, indicating that it is the inability to degrade heparan sulfate and not the specific enzyme deficiency that underlies these defects. As expected, the secondary defects were absent in the brain of an MPS VI mouse, which has no block in heparan sulfate degradation and no accumulated glypican. On the other hand, the weak staining for glypican 1 in MEC of MPS I and MPS II mouse models and the weak or marginal staining for the secondary markers were somewhat surprising since the enzyme deficiencies in MPS I and MPS II result in failure to degrade both heparan sulfate and dermatan sulfate. But we note that although heparan sulfate (the glycan) and glypican (the proteoglycan) are closely related, they are not identical molecules, the proteoglycan being the precursor to the glycan. Perhaps there is some difference in how the proteoglycan in metabolized in MPS I and MPS II compared to MPS III brain.

A second question is why MEC appears to be the most vulnerable area of the brain with respect to these accumulations. It may be due to the heparan sulfate proteoglycan (HSPG) metabolism of MEC. A map of gene expression in the normal mouse brain shows glypican 5 expression to be almost entirely in MEC [37]. In MPS IIIB brain, MEC is enriched in the HSPG glypican 1 and glypican 5, and perhaps some additional HSPG, not yet identified. In the MPS IIIB MEC neurons, some glypican (whether observed by protein or carbohydrate epitopes) may be co-localized with Lamp1 (a marker for membranes of lysosomes and late endosomes), but much is not. The low level of glypican staining in lysosomes can be readily explained by the loss of the protein or carbohydrate epitopes during degradation of the HSPG. On the other hand, the role of the extralysosomal glypican and its subcellular location in the neuron are not known at this time. It may be present on the cell surface, in transport vesicles or in neuronal processes. Some may be present in a biosynthetic compartment, such as the Golgi; a defect of the Golgi has been reported in the brain of MPS IIIB mice [38]. Some HSPG may detach from the membrane and escape into cytoplasm, perhaps from autophagosomes that fail to fuse with lysosomes. Finally, MEC neurons in MPS III may be unable to synthesize sufficient lysosomal membrane to accommodate not only all the partially degraded heparan sulfate, but also all the HSPG that is slated for degradation, causing a backlog of HSPG outside the lysosomes. Some or all of these proposed mechanisms may co-exist. If accumulated HSPG is not sequestered within lysosomes, it could become toxic to the neuron. But since accumulation of glypican is seen only after 2 weeks of postnatal life, it is unlikely to disrupt embryonic or early postnatal brain development.

The toxicity of extralysosomal glypican may have several causes. Since HSPGs bind growth factors and other signaling molecules [39], excessive glypican on the cell membrane would affect some signaling pathways. Second, excessive glypican in a biosynthetic compartment could result in over-modification of the carbohydrate moieties, endowing the carbohydrate chains with some abnormal structure or function. Finally, polyanions such as heparan sulfate and HSPG are known to promote beta amyloid aggregation [40], [41] and tau fiber formation [42]; their presence in a location where they can encounter beta amyloid or Ptau would therefore potentiate the formation of oligomers or larger aggregates of these proteins, which in addition to being toxic, would be difficult to degrade by both lysosomal and proteasomal pathways. Thus the simplest unitary explanation for the pleiotropic effects in MEC of MPS IIIB (and MPS IIIA) mice is that the neurons in that area of the brain accumulate more HSPG than they can sequester in lysosomes, and that the non-lysosomal HSPG causes secondary defects and thereby exacerbates the problems of the underlying storage disease.

## Materials and Methods

### Mouse colony and brain collection

Animal studies were approved by the Chancellor's Animal Research Committee of the University of California Los Angeles, protocol ARC 1994-075. MPS IIIB [2] and MPS I [3] mice were from colonies developed and maintained by us on a C57BL6 background. For brain collection, the mice were euthanized with pentobarbital (100 mg/kg). The brains were removed and post-fixed in 3.65% phosphate-buffered formaldehyde (Fischer Scientific) at 4°C and stored at 4°C until used. In some of the early experiments, the mice were first perfused through the heart as described [22]. Brains from other mouse models were provided by Steven Walkley (MPS IIIA), Joseph Muenzer (MPS II) and Alberto Auricchio (MPS VI). Sections, 40 µm thick, were cut sagittally on a Leica Microsystems Vibratome VT1000S.

### Immunohistochemistry

#### Primary antibodies

These are listed together with the provenance, distributors and catalogue numbers. Glypican 1, rabbit polyclonal, Novus Biologicals NBP1-18666; glypican 2, goat polyclonal, R&D systems AF2355; glypican 5 rabbit polyclonal, Santa Cruz Biotechnology sc84278; heparan sulfate HepSS1 mouse monoclonal IgM, Seikagaku/Amsbio 270426; Δ heparan sulfate F69-3G10, mouse monoclonal IgG, Seikagaku 370260, used with heparitinase (heparan lyase) from *Flavobacterium heparinum,* Seikagaku 100703; PHF tau mouse monoclonal IgG, clone AT100, Endogen MN1060; PHF tau mouse monoclonal IgG, clone AT270, Pierce Endogen MN1050; Gsk3β rabbit polyclonal, GenScript A00196; P-Gsk3β (Y216) rabbit polyclonal, Abcam ab75745; beta amyloid 1-14, rabbit polyclonal, Abcam ab2539; beta amyloid peptide 1–40, mouse monoclonal IgG, Covance SIG-39140; beta amyloid peptide 1–42 mouse monoclonal IgG clone 11A50-B10, Covance SIG-39142; amyloid precursor protein 44–63, goat polyclonal, Abcam ab77994; A11, rabbit polyclonal, Invitrogen AHB0052; LC3, rabbit polyclonal, Novus Biologicals NB100-2331; ubiquitin, rabbit polyclonal, DAKO z0458; ubiquitin (Ubi1, polyubiquitin chains), mouse monoclonal IgG, Abcam ab7254; p62, mouse monoclonal IgG, Novus Biologicals H00008878-M01; GM3 ganglioside, mouse monoclonal IgM, clone GMR6, Seikagaku 370695; nitrotyrosine, rabbit polyclonal, Millipore 06–284; Lamp1, rat monoclonal IgG, clone 1D4B, Santa Cruz SC19992; O-GlNAc, mouse monoclonal IgM (CTD110.6) was provided by Gary Hart [43]; SCMAS, rabbit polyclonal, was commercially prepared for us [20].

#### Secondary antibodies

Biotin-labeled antibodies were obtained from Jackson ImmunoResearch Laboratories: donkey anti-mouse IgG (715-066-150), donkey anti-mouse IgM (715-066-020), donkey anti-rabbit IgG (711-066-152); and from Vector Research Lab, horse anti-goat IgG (BA9500). Fluorescent antibodies were obtained from Invitrogen: AlexaFluor 488 donkey anti- mouse IgG (A21202); AlexaFluor 488 donkey anti-rat IgG, A21208; AlexaFluor 647 donkey anti-rabbit IgG (A31573).

### Sample preparation for light microscopy and confocal microscopy

Sections, 40 µm thick, were cut sagittally on vibratome VT 1000S (Leica microsystems). Slices were permeabilized in 1% (v/v) triton X-100/phosphate-buffered saline (PBS) for 1h at ambient temperature. After washing in the buffer, slices were placed on vectabond-coated glass slides and dried overnight. They were then rehydrated in PBS and washed in ice-cold methanol for 10 min.

For immunohistochemistry, slices were additionally washed in 0.3% (v/v) H_2_O_2_ in methanol at ambient temperature and incubated sequentially with 0.5% (v/v) normal donkey serum, primary antibody and biotin-labeled F(ab')_2_ donkey secondary antibody in Tris-buffered saline (pH 7.5) containing 2% (w/v) bovine serum albumin and 0.02% (v/v) Tween 20 (Tween 20 was excluded when staining for ganglioside GM3). Antigen retrieval by a brief wash in 60% (v/v) formic acid was included prior to staining for Abeta 1–40, Abeta 1–42 and A11. The signal was detected by the Vecstain ABC Elite kit, visualized with diaminobenzidine and counterstained with hematoxylin.

For staining with 3G10 antibody, vibratome sections were first incubated at 37^o^C for 2 h with heparitinase (5 or 10 munit/ml) in 100 mM NaCl, 1 mM CaCl_2_, 50 mM Hepes pH 7.0, 0.01% bovine serum albumin and protease inhibitors (Thomas Scientific #78425), in a total volume of 0.5 ml.

For double immunostaining, the signal was detected by secondary antibodies conjugated to Alexa 488 and Alexa 647 (Invitrogen). Images were acquired by laser scanning confocal microscopy (Pascal 5, Zeiss) and processed by Axiovision software (Carl Zeiss Microimaging) and Adobe Photoshop.

### Lithium treatment

Eight *Naglu −/−* and 8 *Naglu* +/− male mice were fed standard chow (Teklad 7013) containing 3 g/kg LiCl, purchased from Harlan Laboratories; 8 *Naglu −/−* and 8 *Naglu*+/*−* male mice were fed the standard chow. The LiCl chow contained 0.05 g/kg blue food coloring for easy identification. Since lithium might cause loss of sodium, the mice receiving lithium were provided with a small bottle of water containing 0.9% NaCl in addition to their regular water supply; however, they used little if any of the saline supplement. To get the pups used to the taste of LiCl, the experiment was started at P12, with the mother receiving chow containing 2 g/kg LiCl, and the pups were switched to the 3 g/kg regimen upon weaning (P20). The mice were sacrificed at P89–92 for examination of brain pathology.
