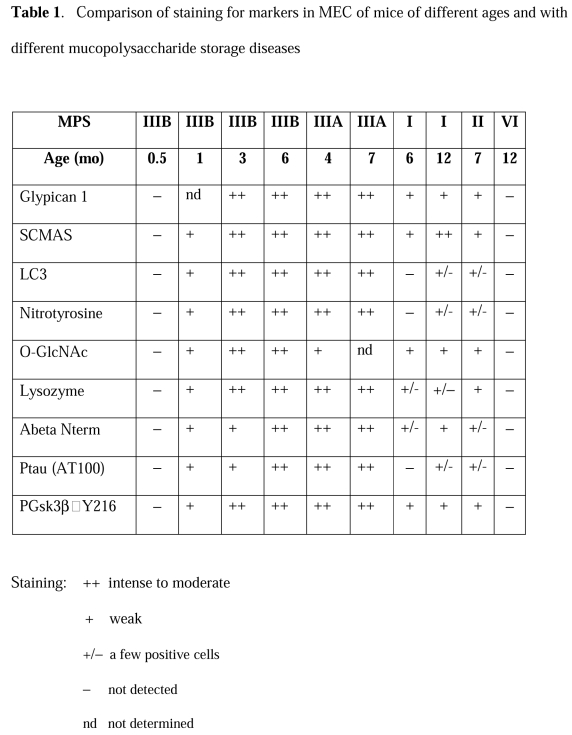# Correction: Defects in the Medial Entorhinal Cortex and Dentate Gyrus in the Mouse Model of Sanfilippo Syndrome Type B

**DOI:** 10.1371/annotation/3de78cba-af11-4deb-939a-bf0803592d08

**Published:** 2012-01-26

**Authors:** Kazuhiro Ohmi, Hui-Zhi Zhao, Elizabeth F. Neufeld

Table 1 is incomplete. The correct Table 1 can be viewed here: 

**Figure:**